# Moisture Absorption Effects on Mode II Delamination of Carbon/Epoxy Composites

**DOI:** 10.3390/polym12092162

**Published:** 2020-09-22

**Authors:** King Jye Wong, Mahzan Johar, Seyed Saeid Rahimian Koloor, Michal Petrů, Mohd Nasir Tamin

**Affiliations:** 1 School of Mechanical Engineering, Faculty of Engineering, Universiti Teknologi Malaysia, Johor Bahru 81310, Malaysia; nasirtamin@utm.my; 2 Faculty of Engineering and Science, Curtin University Malaysia, Miri 98009, Malaysia; mahzan.johar@curtin.edu.my; 3 Institute for Nanomaterials, Advanced Technologies and Innovation (CXI), Technical University of Liberec (TUL), Studentska 2, 461 17 Liberec, Czech Republic; s.s.r.koloor@gmail.com (S.S.R.K.); michal.petru@tul.cz (M.P.); 4 Department of Aerospace Engineering, Faculty of Engineering, Universiti Putra Malaysia, Serdang 43400, Malaysia

**Keywords:** carbon/epoxy composite, moisture absorption, Fickian, delamination, fracture toughness, cohesive zone modeling

## Abstract

It is necessary to consider the influence of moisture damage on the interlaminar fracture toughness for composite structures that are used for outdoor applications. However, the studies on the progressive variation of the fracture toughness as a function of moisture content *M* (%) is rather limited. In this regard, this study focuses on the characterization of mode II delamination of carbon/epoxy composites conditioned at 70 °C/85% relative humidity (RH). End-notched flexure test is conducted for specimens aged at various moisture absorption levels. Experimental results reveal that mode II fracture toughness degrades with the moisture content, with a maximum of 23% decrement. A residual property model is used to predict the variation of the fracture toughness with the moisture content. Through numerical simulations, it is found that the approaches used to estimate the lamina and cohesive properties are suitable to obtain reliable simulation results. In addition, the damage initiation is noticed during the early loading stage; however, the complete damage is only observed when the numerical peak load is achieved. Results from the present research could serve as guidelines to predict the residual properties and simulate the mode II delamination behavior under moisture attack.

## 1. Introduction

Carbon/epoxy composites are increasingly used in aircraft industry [1,2]. Nevertheless, aircraft structures are exposed to different environmental conditions throughout their service lifetime. It is commonly reported that moisture absorption could lead to the degradation in the mechanical properties of composite materials [3,4,5,6]. Not only that, as the external loadings in real life applications are generally induced in the lateral direction, mode II delamination could be dominant due to the interlaminar shear stress caused by the bending of the structures [7]. Mode II delamination refers to the separation of the neighboring plies in the composite due to the shearing effect between the plies. Therefore, it is essential to investigate the moisture absorption effects on the mode II delamination behavior of composite laminates.

LeBlanc and LaPlante [8] reported that upon distilled water immersion at aging temperature of 70 °C for around 11 months, the mode II fracture toughness *G_IIC_* of the carbon/epoxy composites has decreased by 37.5% when compared to the unaged specimens. Davidson et al. [9] conditioned the thermoplastic particles toughened carbon/epoxy composites at 50 °C/95%RH for approximately 11 months as well. The *G_IIC_* was found to have deteriorated by around 20% when the specimens were tested in environments of –43 °C and 98 °C. As for eight-harness satin-weave glass/epoxy composites immersed in distilled water at aging temperature of 72 °C, *G_IIC_* has dropped by 55% and 40% in warp and weft directions respectively after around 4 months of immersion [10]. Zhao et al. [11] aged eight-harness satin-weave glass/bismaleimide composites in seawater for around 8 months. The authors discovered that Teflon insert specimens showed degradation in *G_IIC_* at aging temperatures of 50 °C and 80 °C for 22% and 32%, respectively. However, for precracked specimens, *G_IIC_* decreased by 18% at 50 °C but increased by 19% at 80 °C. Nash et al. [12] immersed non-crimp carbon fabric/benzoxazine composites in deionized water at aging temperature of 80 °C for nearly 4 months. Two types of benzoxazine were used, which were toughened (BZ9120) and untoughened (BZ9130) resins. In addition, two types of crack tip conditions were considered, which were non-precracked and precracked specimens. For non-precracked specimens, the authors discovered *G_IIC_* increased by 153% and 34% for BZ9120 and BZ9130 resins, respectively. As for precracked specimens, the authors found invariant *G_IIC_* for both resins.

From the above literature, it is apparent that the moisture effects on the mode II delamination is still not conclusive. In addition, characterization was mainly conducted at dry and saturated conditions, and hence the variation of the *G_IIC_* throughout the aging period was not known. Recently, it has been demonstrated that mode I, II, and mixed-mode I/II fracture toughness varied at a different rate with respect to the moisture content [13]. Nevertheless, the study was limited to distilled water immersion. For aircraft applications, the highest humidity level is commonly taken as 85% relative humidity (RH) [14].

In this research, moisture effects on the mode II delamination of unidirectional carbon/epoxy composites were studied. The composites were continuously conditioned at 70 °C/85%RH and tested using three-point end-notched flexure (ENF) test at various moisture content levels. The mode II fracture toughness results were fitted using a residual property model. This was followed by the numerical analyses of the mode II delamination behavior using cohesive zone modeling.

## 2. Materials and Methods

### 2.1. Materials and Specimens

The unidirectional carbon/epoxy prepreg has a nominal thickness of 0.15 mm with an average fiber volume fraction of 65.7% ± 6.3% [15]. All specimens were supplied by X Plas Singapore (Kallang Way, Singapore). Firstly, a 20-ply unidirectional composite plate was manufactured using hand lay-up technique. A 15 μm thick Teflon film was placed at the mid-thickness location to generate the pre-crack. The composite laminate was cured using hot-press technique and cut into specimens of 20 mm width after that.

### 2.2. Moisture Absorption Test

The edges of all specimens were painted with a layer of white water-proof paint. Firstly, this was to minimize the moisture penetration through edges. Secondly, white paint helped in visual inspection of crack propagation during testing. After that, the initial weight of all the specimens were measured using Shimadzu ATY224 four-decimal digital balance (Shimadzu Corp., Kyoto, Japan). They were then placed in Memmert HCP50 (Memmert GmbH + Co. KG, Schawabach, Germany) humidity chamber conditioned at 70 °C/85%RH. The weight gain of the specimens was measured periodically. Three measurements were made to get an average moisture content, *M*.

### 2.3. Mode II Delamination Test

**Figure 1:**
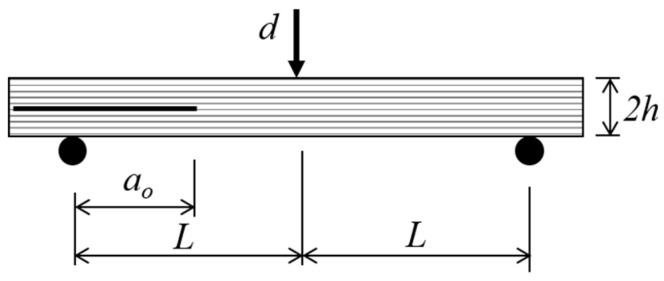
Schematic diagram of ENF test setup.

Mode II delamination test was performed using end-notched flexure (ENF) test (Figure 1) according to ASTM D7905 [16]. The specimen’s thickness *2h* = 3 mm, initial crack length *a_o_* = 30 mm and half span length *L* = 50 mm. Specimens were taken out from the chamber to perform delamination tests at aging intervals of 4, 12, 27, and 156 days, which corresponded to the moisture content *M* = 0.23%, 0.42%, 0.61%, and 0.94%. Delamination tests were also carried out for specimens at dry condition. All tests were performed using a Shimadzu Universal Testing Machine (Shimadzu Corp., Kyoto, Japan) with the load cell capacity of 10 kN at 1 mm/min. At least three specimens were tested for each aging interval. All tests were performed at the ambient conditions.

### 2.4. Date Reduction Scheme

Mode II fracture toughness *G_IIC_* was calculated using Irwin–Kies [17] equation

(1) $$G_{IIC}=\frac{P_{C}^{2}}{2B}\left(\frac{dC}{da}\right)$$

where *P_C_* is the critical load, *B* is the width of the specimen, *C* is the compliance and *a* is the crack length. The compliance calibration model is described by [16]

(2) $$C=C_{2}a^{3}+C_{1}$$

*C*_2_ and *C*_1_ are constants fitted through *C* − *a*^3^ plot. Substituting the derivative of Equation (2) into Equation (1) gives

(3) $$G_{IIC}=\frac{P_{C}^{2}}{2B}\cdot 3C_{2}a_{}^{2}$$

The initial crack length was 30 mm for all moisture content levels. To generate the compliance plot at each moisture content level, additional tests were conducted at crack lengths of 20, 25, 35, and 40 mm within the linear elastic region of the force–displacement curves.

## 3. Experimental Results and Discussion

### 3.1. Moisture Absorption Curves

**Figure 2:**
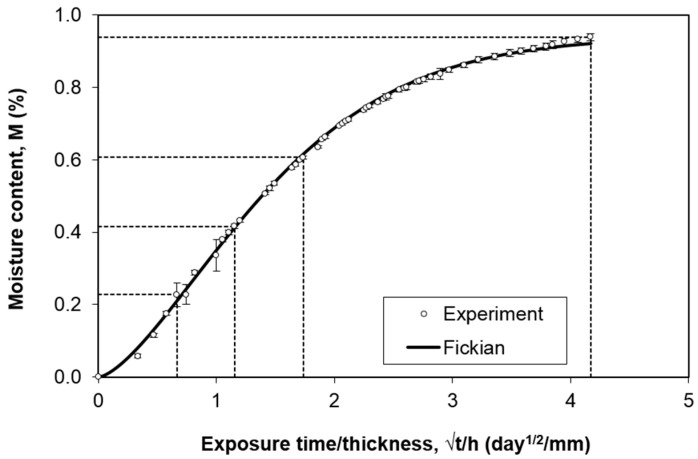
Experimental and fitted moisture absorption curves of the carbon–epoxy composites.

**Figure 3:**
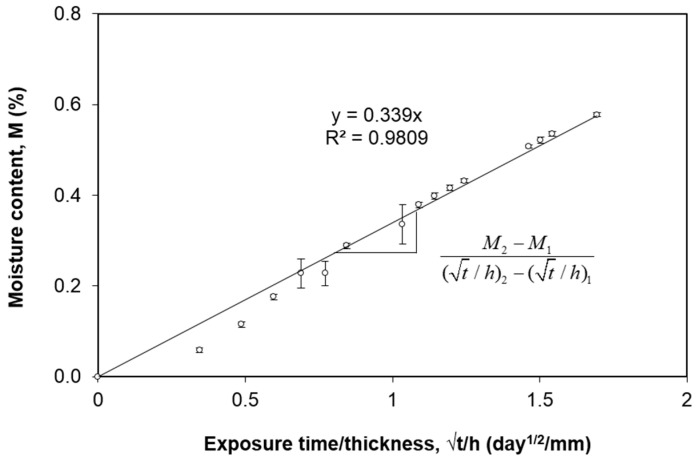
Initial linear region of the moisture content graph.

The average experimental moisture absorption curve is shown in Figure 2. The error bars indicate the standard deviation of each measurement. The average maximum moisture content *M_m_* is 0.94%. To apply Fickian diffusion model [18], the slope of the initial linear region which is up to 60% of *M_m_* is fitted (Figure 3). The value of the slope (0.339) corresponds to $\left(M_{2}-M_{1}\right)/\left[{\left(\sqrt{t}/h\right)}_{2}-{\left(\sqrt{t}/h\right)}_{1}\right]\;$ as described in Equation (4). The diffusivity *D_z_* = 2.56 × 10^−2^ mm^2^/day is thus obtained using Equation (4).

(4) $$D_{z}=\frac{\pi }{{\left(4M_{m}\right)}^{2}}{\left(\frac{M_{2}-M_{1}}{{\left(\sqrt{t}/h\right)}_{2}-{\left(\sqrt{t}/h\right)}_{1}}\right)}^{2}$$

Subsequently, the Fickian diffusion curve is plotted using Equation (5), where *t* indicates the exposure time at any instant. From Figure 2, it could be seen that the Fickian model fits well the experimental data.

(5) $$M_{F}(t)=M_{m}\left\{1-\exp\left[-7.3{\left(\frac{D_{z}t}{h_{}^{2}}\right)}^{0.75}\right]\right\}$$

### 3.2. Mode II Fracture Toughness

**Figure 4:**
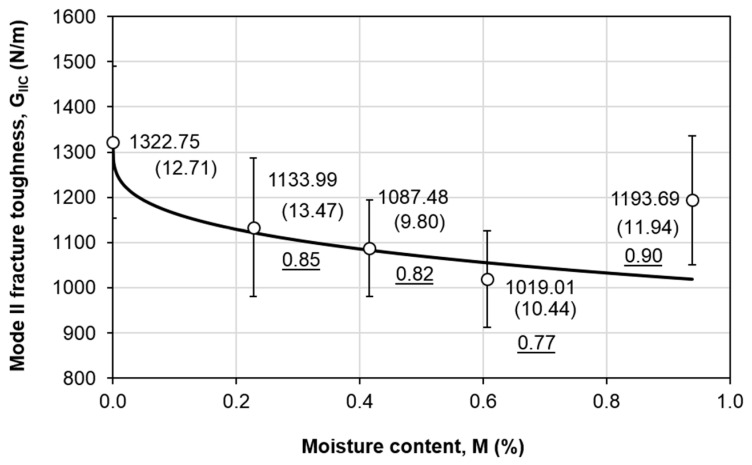
Distribution of mode II fracture toughness with moisture content level.

Figure 4 plots the mode II fracture toughness *G_IIC_* values at different moisture levels. The *G_IIC_* values are calculated using Equation (3), where the *C*_2_ values at different moisture content levels are displayed in Table 1. The maximum coefficient of variation (C.V) is 14% at *M* = 0.23%, which signifies a comparatively good repeatability of the tests. It is noticed that in general *G_IIC_* has been decreased upon moisture attack. In average, the *G_IIC_* values dropped by 15%, 18%, 23%, and 10% at *M* = 0.23%, 0.42%, 0.61%, and 0.94%, respectively. This could be due to matrix degradation that leads to interface weakening effect [11], and is commonly noticed as a result of moisture absorption [4,5,13,19,20,21,22]. The slight increment of the *G_IIC_* at *M* = 0.94% could be due to ductility enhancement due to matrix plasticization [12], which could have enlarged the fracture process zone at the interface [11]. It is worth noting that the matrix plasticization effect is also reflected in the global bending behavior of the composite, which is shown in Figure 6. Matrix plasticization leads to the softening of the material, and hence a lower stiffness is observed upon moisture absorption. Nevertheless, overall, it is apparent that moisture has degraded the mode II fracture toughness. Compared to the previous study on the water absorption effects on another type of unidirectional carbon/epoxy composite by immersion in distilled water at 70 °C, it is apparent that distilled water has a more severe effect than humidity. The maximum moisture content *M*_m_ was 5.3%, which was accompanied by a drop of approximately 50% in the *G_IIC_* as compared to the dry specimens [13].

### 3.3. Force–Displacement Curves

**Figure 5:**
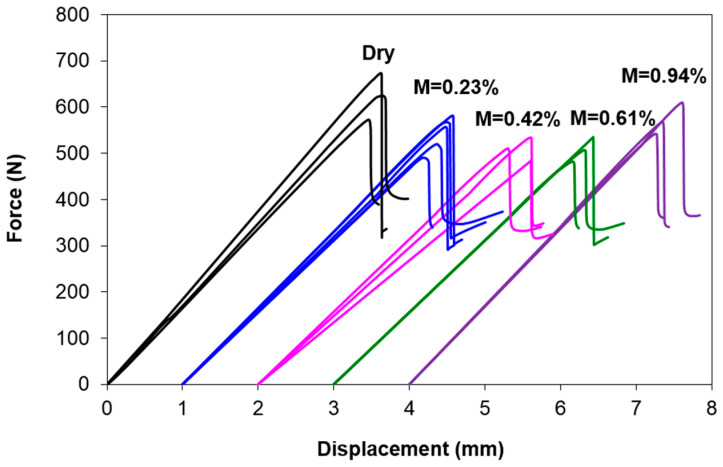
Force–displacement curves of ENF specimens at various moisture content levels.

**Figure 6:**
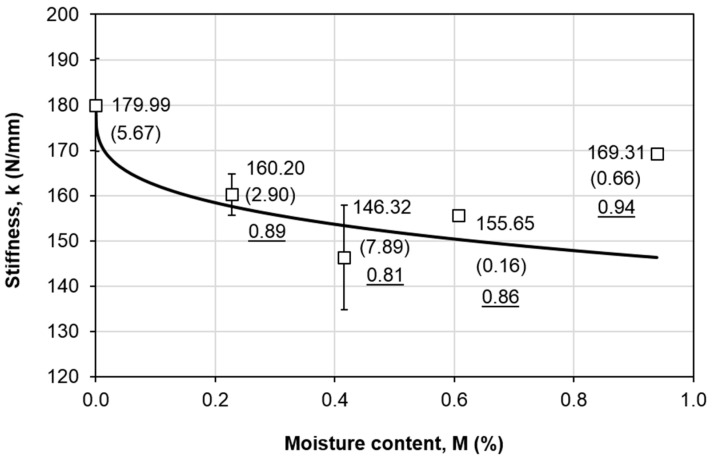
Variation of the stiffness with respect to the moisture content level.

Figure 5 shows the force-displacement curves at various moisture content levels. Upon loading, the force increases linearly with the displacement. When the maximum force is attained, abrupt force drop is noticed for all force-displacement curves. This signifies unstable crack propagation. Figure 6 shows that the average slopes (initial linear region of the force-displacement curves) are 179.99, 160.20, 146.32, 155.65, and 169.31 N/mm at *M* = 0%, 0.23%, 0.42%, 0.61%, and 0.94%, respectively (marked as dotted lines in Figure 2). The values in bracket indicate the coefficient of variation (C.V) in percentage. The largest C.V is 7.89% for *M* = 0.42%, which partially implies a good repetition of the specimens and the tests. The underlined values refer to the ratio of the corresponding stiffness with respect to the stiffness at dry condition. In overall, the average stiffness of all wet specimens is lower than the dry specimens, despite the trend is not consistent. The average stiffness decreases with the moisture content until *M* = 0.42% (with a 19% decrement), and a slight increment is observed after that. Further explanation will be discussed in Section 3.3.

### 3.4. Residual Property Model

To describe the variation of *G_IIC_* with respect to moisture content *M*, the following residual property model (RPM) [3,6,13] is adopted

(6) $$G_{IIC}(M)=G_{IIC,dry}\left[1-\left(1-\frac{G_{IIC,\min}}{G_{IIC,dry}}\right){\left(\frac{M}{M_{m}}\right)}^{\zeta }\right]$$

where *G_IIC_(M)* is the residual mode II fracture toughness at particular moisture content, *G_IIC,dry_* is the dry property, *G_IIC,min_* is lowest mode II fracture toughness within the range of study, *M* is the moisture content, *M*_m_ is the maximum moisture content, and *ζ* is the degradation parameter. This model assumes that the residual mode II fracture toughness is a function of moisture content only. The best-fit curve with *ζ* = 0.2918 is plotted as the solid line in Figure 4. The largest difference is found at *M* = 0.94%, with a 15% difference. This is due to the fluctuation in the trend, where a slight increment in the *G_IIC_* is noticed towards the end of the aging period. Nevertheless, the predicted *G_IIC_* is more conservative (lower than the experimental value), thus a lower predicted value with 15% difference is acceptable from the safety point of view. The same model is then applied for the stiffness (Figure 6), which is written as

(7) $$k(M)=k_{dry}\left[1-\left(1-\frac{k_{\min}}{k_{dry}}\right){\left(\frac{M}{M_{m}}\right)}^{\zeta }\right]$$

**Figure 7:**
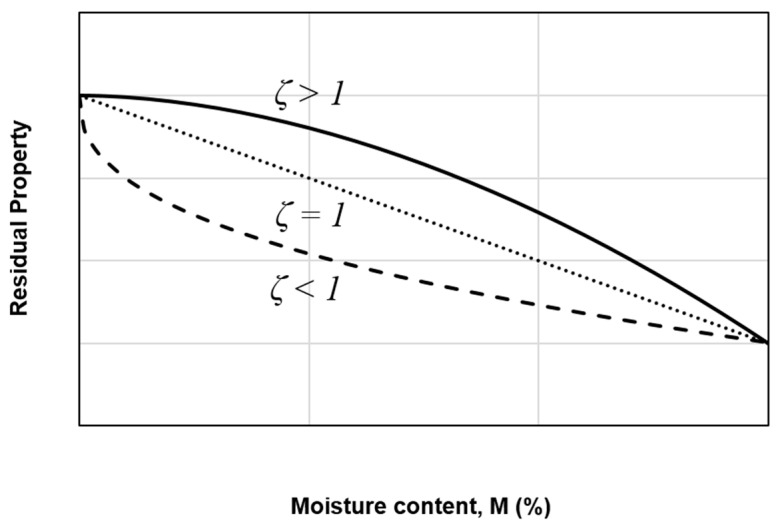
Three possible degradation trends of the residual property with respect to the moisture content.

A similar best-fit parameter is obtained, with *ζ* = 0.2877, and the largest difference is also found at *M* = 0.94%, with a 14% difference. This illustrates that both stiffness and mode II fracture toughness follow the similar degradation trend. A *ζ* value below unity implies that corresponding property is sensitive to moisture attack. As described in Figure 7. on the three possible degradation trends, *ζ* > 1 depicts an initial stable value, *ζ* < 1 indicates a significant drop during the early aging period, and *ζ* = 1 signifies a linear degradation.

## 4. Numerical Simulation

### 4.1. Finite Element Model

**Figure 8:**
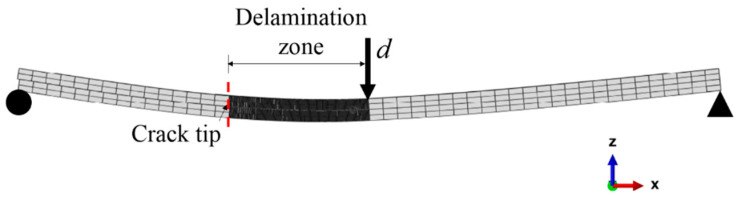
Finite element model of the ENF specimen.

Figure 8 illustrates the finite element model of the ENF specimen. The boundary conditions are roller on one side and pinned on another end. A vertical downward displacement is applied at the middle of the model. Composite arms are modeled using continuum shell elements (SC8R), while the mid-plane interface is modeled using cohesive elements (COH3D8). The entire composite is meshed by four elements in the thickness direction, which has shown to be enough to simulate the bending behavior of the composite [15]. To avoid interpenetration between the upper and lower arms, contact surfaces are defined at the adjacent surfaces of the interface using frictionless contact. An element thickness of 10 µm is used for the cohesive elements [15,23], which is also in the same order of the resin-rich region [24] and the Teflon thickness. The delamination zone is meshed with 0.1 mm element size, while the region outside the delamination zone is meshed with mesh size of 2 mm. Across the width, the specimen is discretized by an element size of 0.5 mm.

### 4.2. Lamina Properties

Firstly, the back-calculated modulus *E_f_* of each specimen is calculated using the equation

(8) $$E_{f}=\frac{3a_{o}^{}{}^{3}+2L^{3}}{8Bh^{3}C}$$

The average *E_f_* at each moisture content is listed in the second column of Table 2. It noteworthy that, for unidirectional laminates, *E_f_* is equal to the longitudinal modulus *E*_11_. The values in bracket indicate the C.V(%). Good repeatability is found, with a maximum C.V of less than 8%. The *E*_11_ value estimated at dry condition is the same as reported in a previous study [15], where the same carbon/epoxy composite was used. Therefore, the same lamina properties reported in reference [15] are used for dry condition. As for the other lamina properties (transverse modulus *E*_22_, in-plane shear modulus *G*_12_, out-of-plane shear modulus *G*_13_, and *G*_23_) at wet conditions, they are estimated using the same ratio of the corresponding *E*_11_ with respect to its dry value. The Poisson’s ratio (*ν*_12_) is assumed to be invariant with the moisture content [6]. It is to note that the transverse and shear properties are generally recognized to be sensitive to moisture attack [6]. However, since the bending behavior of the composite is dominated by *E*_11_, the accuracy of the transverse and shear properties are therefore not critical in this case.

### 4.3. Cohesive Properties

**Figure 9:**
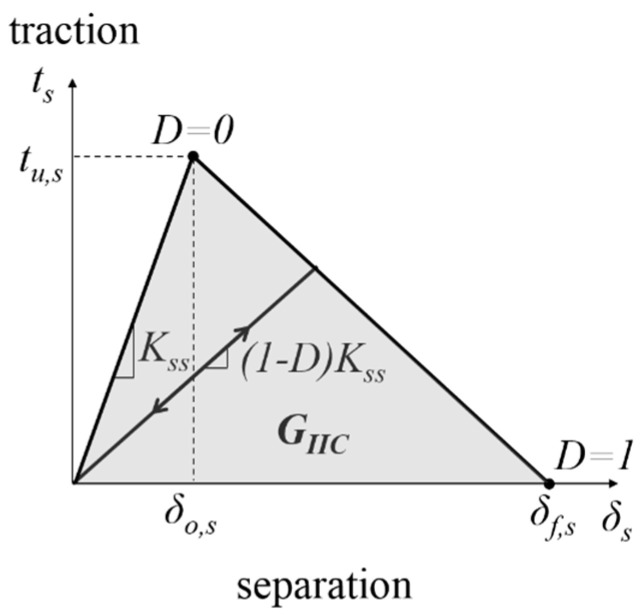
Schematic diagram of the bilinear traction separation law.

In this study, bilinear traction-separation law is adopted (Figure 9). This law is commonly used due its simplicity and accuracy [15,25,26]. Upon loading, the traction increases linearly with the separation. When the interface shear strength *t_u,s_* is reached, damage is initiated (*D* = 0). Further increment in the separation results in the traction decrement, which signifies softening effect. When the traction is reduced to 0, the element is completely failed (*D* = 1). The area under the traction–separation curve corresponds to the mode II fracture toughness *G_IIC_*.

As shown in Figure 9, the input parameters for the cohesive zone model subjected to mode II shear loading are interface shear stiffness *K_ss_*, interface shear strength *t_u,s_*, and the mode II fracture toughness *G_IIC_*. *G_IIC_* is determined from the experiments or estimated using Equation (6). *K_ss_* at the dry condition is assumed to be 4.5 × 10^5^ MPa/mm, which is the same value as the interface normal stiffness *K_nn_* used to simulate mode I delamination of the same composite [15]. It is common that the same normal and shear interface stiffness value is used for the same material [27].

The interface shear strength at dry condition is estimated using Equation (9) [28]

(9) $$t_{u,s}=\sqrt{\left(\frac{G_{IIC}}{G_{IC}}\right)\left(\frac{K_{ss}}{K_{nn}}\right)}t_{u,n}$$

In Equation (9), *G_IC_* is the mode I fracture toughness and *t_u,n_* refers to the interface normal strength. In a separate study on mode I delamination of the same material, it was reported that *G_IC_* = 245 N/m at quasi-static loading. In addition, the interface normal strength *t_u,n_* = 35 MPa was found to be a good choice to obtain reliable simulation results [15]. Using Equation (9), the value of *t_u,s_* at dry condition is estimated to be 81 MPa.

The interface shear stiffness at wet condition is estimated using the same trend as described by Equation (7), where

(10) $$K_{ss}(M)=K_{ss,}{}_{dry}\left[1-0.1871{\left(\frac{M}{M_{m}}\right)}^{0.2877}\right]$$

Using the same approach, the interface shear strength at wet condition is estimated using

(11) $$t_{u,s}(M)=\sqrt{\left(\frac{G_{IIC}(M)}{G_{IIC,dry}}\right)\left(\frac{K_{ss}(M)}{K_{ss,dry}}\right)}t_{u,s,dry}$$

The cohesive parameters used at various moisture content levels are listed in Table 3. It is noteworthy that the *G_IIC_* are the estimated values from Equation (6) instead of the experimental values. The intention is to evaluate the accuracy of the predicated *K_ss_(M)* and *G_IIC_(M)* using the RPM.

### 4.4. Simulation Results

**Figure 10:**
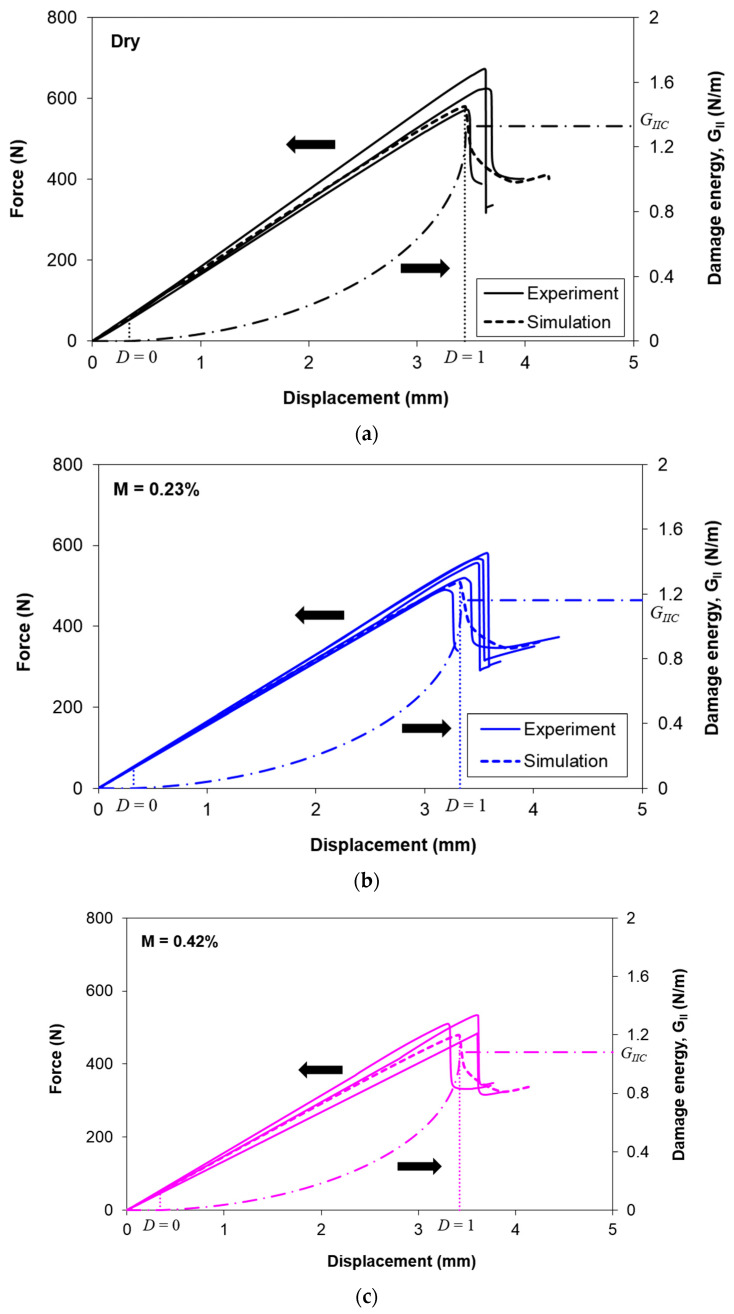

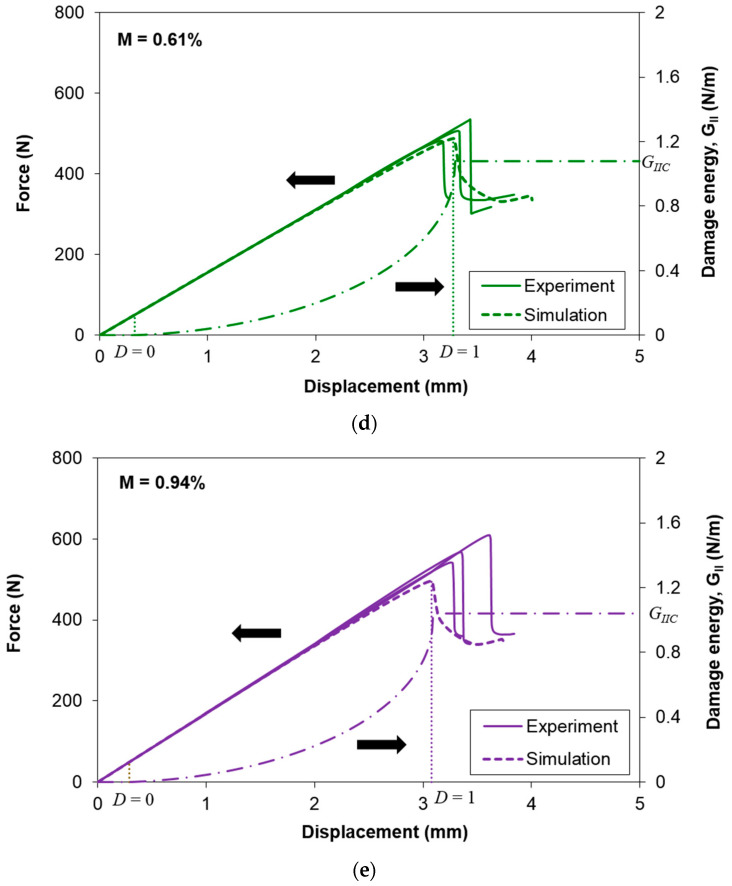
Experimental and numerical force displacement curves at moisture content of (**a**) 0%, (**b**) 0.23%, (**c**) 0.42%, (**d**) 0.61%, and (**e**) 0.94%, along with the evolution of the damage energy.

Figure 10 compares the experimental and numerical force displacement curves at each moisture content level. The maximum difference between the experimental and numerical slopes is 10%, which is found in dry conditions. This implies that the approach to estimate the elastic properties of the lamina as described in Section 4.2 is reliable. In addition, the difference between the experimental and numerical peak load is less than 12.5%, except for the case at *M* = 0.94%, where 18.5% is attained. This is because the *G_IIC_* values used in the simulation are the predicted values using Equation (6), and it is shown in Figure 4 that the fitted *G_IIC_* value at *M* = 0.94% is 15% lower than the experimental value. Therefore, it is reasonable to observe a larger difference in the peak load.

In addition, it is found that for all cases, the first element that experiences damage is at the crack tip of the mid-width location. The damage is initiated (*D* = 0 indicated in Figure 9 and Figure 10) during the early stage of loading (approximately 0.3 mm of crosshead displacement). Beyond that, the mode II damage energy is developed, which is represented by the area under the curve of the traction-separation law (Figure 9). When the element is totally damaged (*D* = 1 indicated in Figure 9 and Figure 10), it also corresponds to the instant where the numerical peak load is attained.

## 5. Conclusions

In this study, mode II delamination behavior of unidirectional carbon/epoxy composites subjected to moisture exposure of 70 °C/85%RH was studied using end-notched flexure test. The mode II fracture toughness at moisture content levels of 0%, 0.23%, 0.42%, 0.61%, and 0.94% were determined and fitted using a residual property model. The mode II delamination behavior was also simulated using cohesive elements. Based on the results, it can be concluded that:

- The moisture absorption is well fitted using Fick’s law, with the maximum moisture content of approximately 0.94% and diffusivity of 2.56 × 10^−2^ mm^2^/day.
- In general, mode II fracture toughness decreases with the moisture content. The maximum degradation is 23% at moisture content of 0.61%.
- The variation of the mode II fracture toughness is well fitted using the residual property model, with a 15% difference at the moisture content of 0.94%.
- The maximum difference between the experimental and numerical slopes is 10% under dry conditions. This signifies that the approach of estimating the lamina properties and interface shear stiffness used in this study is reliable.
- The difference between the experimental and numerical peak loads is less than 12.5%, except for the case at maximum moisture content of 0.94%. This indicates that the mode II fracture toughness values predicted using the residual property model and the methodology adopted to estimate the interface shear strength provide sufficient accuracy in predicting the mode II delamination behavior of the composite.
- The numerical results show that the damage is initiated in the interface during the early stage of loading. When the numerical peak load is attained, the first element has reached its total failure.

## Figures and Tables

**Table 1:** Compliance constant at different moisture content levels.

Moisture Content *M* (%)	*C*_2_ (×10^−8^ N^−1^mm^−2^)
0	5.57
0.23	5.65
0.42	6.18
0.61	5.82
0.94	5.35

**Table 2:** Lamina properties for the carbon/epoxy composite used in this study [15].

*M* (%)	*E*_11_ (GPa)	*E*_22_ (GPa)	*G*_12_ (GPa)	*G*_13_ (GPa)	*G*_23_ (GPa)	*ν*_12_
0	103 (4.95)	6.7	2.7	2.7	2.5	0.34
0.23	94 (2.90)	6.1	2.4	2.4	2.3	0.34
0.42	86 (7.89)	5.5	2.2	2.2	2.1	0.34
0.61	91 (0.16)	5.9	2.4	2.4	2.2	0.34
0.94	99 (0.66)	6.4	2.6	2.6	2.4	0.34

**Table 3:** Cohesive parameters used for different moisture content levels.

*M* (%)	*G_IIC_* (N/m)	*K_ss_* (MPa/mm)	*t_u,s_* (MPa)
0	1322.75	4.50 × 10^5^	81
0.23	1121.90	3.94 × 10^4^	70
0.42	1083.24	3.83 × 10^4^	68
0.61	1055.34	3.76 × 10^4^	66
0.94	1019.01	3.66 × 10^4^	64
